# In Cellulo and In Vivo Comparison of Cholesterol, Beta-Sitosterol and Dioleylphosphatidylethanolamine for Lipid Nanoparticle Formulation of mRNA

**DOI:** 10.3390/nano12142446

**Published:** 2022-07-17

**Authors:** Ayoub Medjmedj, Albert Ngalle-Loth, Rudy Clemençon, Josef Hamacek, Chantal Pichon, Federico Perche

**Affiliations:** 1 Centre de Biophysique Moléculaire, UPR4301 CNRS, Rue Charles Sadron, 45071 Orléans, France; ayoub.medjmedj@cnrs-orleans.fr (A.M.); albert.ngalle-loth@cnrs-orleans.fr (A.N.-L.); rudy.clemencon@cnrs.fr (R.C.); josef.hamacek@cnrs.fr (J.H.); pichon@cnrs.fr (C.P.); 2 Centre de Biophysique Moléculaire, University of Orléans, 45100 Orléans, France

**Keywords:** mRNA therapy, intracellular trafficking, LNP

## Abstract

Lipid Nanoparticles (LNPs) are a leading class of mRNA delivery systems. LNPs are made of an ionizable lipid, a polyethyleneglycol (PEG)-lipid conjugate and helper lipids. The success of LNPs is due to proprietary ionizable lipids and appropriate helper lipids. Using a benchmark lipid (D-Lin-MC3) we compared the ability of three helper lipids to transfect dendritic cells in cellulo and in vivo. Studies revealed that the choice of helper lipid does not influence the transfection efficiency of immortalized cells but, LNPs prepared with DOPE (dioleylphosphatidylethanolamine) and β-sitosterol were more efficient for mRNA transfection in murine dendritic cells than LNPs containing DSPC (distearoylphosphatidylcholine). This higher potency of DOPE and β-sitosterol LNPs for mRNA expression was also evident in vivo but only at low mRNA doses. Overall, these data provide valuable insight for the design of novel mRNA LNP vaccines.

## 1. Introduction

Messenger RNA (mRNA) is a promising means of vaccination [1,2,3,4,5,6,7,8]. The potency of mRNA technology to rapidly provide approved vaccines has recently been demonstrated with anti-SARS-CoV-2 vaccines [9]. These approved mRNA vaccines are formulated as Lipid nanoparticles (LNP), the most clinically advanced mRNA delivery system, to protect mRNA against nucleases and allow intracellular uptake and translation of the mRNA [10,11,12]. In vivo, mRNA should be delivered to antigen-presenting cells such as dendritic cells (DCs) to induce adaptive protective immune responses [6,10,13].

LNPs are prepared by microfluidic mixing of lipids in ethanol and mRNA in an acidic buffer (pH ≤ 4.0) [11]. Lipids include an ionizable lipid (pKa < 7) that will be protonated at acidic pH to condense mRNA and release mRNA inside the cells. This multicomponent system also contains cholesterol for stabilization, a helper lipid for endosomal escape (DOPE (1,2-dioleoyl-sn-glycero-3-phosphatidyl-ethanolamine) or DSPC (distearoylphosphatidylcholine) and a PEGylated (polyethyleneglycol) lipid to prevent aggregation of LNPs. Moreover, several techniques exist for the large scale production of mRNA LNPs [14].

During the endocytosis of nanoparticles (including LNPs), the cell plasma membrane starts to invaginate to entrap nanoparticles inside small vesicles. These vesicles are routed to the early endosome and, subjected to an acidic pH gradient from mildly acid early endosomes (pH 6.3) to late endosomes (pH 5.5) and finally lysosomes (pH < 5) [15,16,17]. As lysosomes are a degradation compartment, a key parameter for a LNP formulation is to limit mRNA entrapment in the endosomes (reviewed in [4,10,11,12]). Indeed, only ≈1–2.5% mRNA was detected in the cytosol after transfection of human epithelial cells with mRNA LNPs made of the ionizable lipid D-Lin-MC3-DMA, which is commonly used for those formulations mRNA LNPs [18,19]. This drastic limiting step led to the development of formulations with different strategies for enhanced intracellular delivery and endosomal escape [12].

Previous studies with mRNA LNPs insisted on the importance of the helper lipid or the cholesterol analog for mRNA transfection [11,20,21,22]. The flexibility of mRNA, with an exposition of nucleobases leads to electrostatic and hydrophobic interactions with the lipids, similar to single-stranded DNA [23,24]. Structures of mRNA-LNPs have been described by cryo-TEM (Transmission electron cryomicroscopy) and SAXS (Small-Angle X-ray Scattering) either as lamellar [25] or disordered inverted hexagonal [26]. The influence of lipid composition on LNP structure has been reviewed recently, with a focus on resolved structures [27]. SAXS studies identified distinct locations of lipids in LNPs, with segregation of DSPC at their surface [25]. Moreover, the choice of helper lipid was shown to drastically change LNP tissue tropism after intravenous injection and in vivo mRNA expression due to the binding of distinct proteins in the circulation [28,29]. Concerning the helper lipid, DOPE was shown to yield higher cellular transfection efficiency over the more frequently used DPSC with less inflammation at the injection site [30,31,32,33]. This was attributed to the organization of DOPE in the hexagonal phase favorable to membrane fusion [22,34]. The Sahay group revealed higher potency of LNPs prepared with the cholesterol analog β-sitosterol [21,35]. The inclusion of β-sitosterol instead of cholesterol improved mRNA transfection by 48-fold in cancer cells and by 14-fold in primary macrophages. 

Such enhancement was attributed to differences in LNPs microstructure and intracellular trafficking.

Studies on helper lipids were carried on by different groups, making side-by-side comparisons difficult. We used a FDA-approved lipid formulation of D-Lin-MC3, cholesterol, DSPC and DMG-PEG at 50:38.5:10:1.5 molar ratios as a benchmark. This formulation is approved as the siRNA therapy ONPATTRO^®^ [36]. Aiming to present correlations for LNP performance, we compared three types of LNPs containing either DSPC and cholesterol, DOPE and cholesterol or DOPE and β-sitosterol based on their ability to transfect cell lines in culture, their intracellular trafficking patterns in DC, and in vivo expression after intramuscular injection.

## 2. Materials and Methods

All reagents were purchased from Sigma (St. Quentin Fallavier, France) unless otherwise stated. mMESSAGE mMACHINE T7 ULTRA Transcription Kit, was purchased from Thermo Fisher (Montigny-le-Bretonneux, France). DOPE (1,2-Dioleoyl-sn-glycero-3-phosphoethanolamine), DMG-PEG (1,2-dimyristoyl-rac-glycero-3-methoxypolyethylene glycol-2000), were from Avanti Polar Lipids (Alabaster, AL, USA). DSPC (1,2-distearoyl-sn-glycero-3-phosphocholine), cholesterol and β-Sitosterol (ref 85451) were from Sigma Aldrich (Lyon, France). D-Lin-MC3 was from Clinisciences (Nanterre, France). All primers were obtained from Eurogentec (Seraing, Belgium). The structures of these lipids are presented in Figure S1.

### 2.1. Plasmids

The pGEM4Z-EGFP plasmid containing a T7 promoter for the production of EGFP RNA has been previously described [37,38]. GFP-rab7 WT was a gift from Richard Pagano (Addgene plasmid #12605) [39]. EGFP-Rab11a-7 was a gift from Michael Davidson (Addgene plasmid #56444) [40]. pDNA used in this study was amplified in E. coli DH5α and purified using an Endofree Plasmid Mega Kit (Qiagen, Courtaboeuf, France).

### 2.2. In Vitro Transcription of GFP mRNA

Anti-reverse cap analog (ARCA)-capped RNA with a poly(A) tail coding the reporter gene EGFP (Enhanced Green Fluorescent Protein) was produced by in vitro transcription using the T7 mMessage mMachine kit as in [37,38]. The RNA concentration was determined by absorbance at 260 nm; RNA had 260:280 ratios ≥ 2 and was stored at −80 °C in small aliquots. The quality of GFP mRNAs was verified by Agilent analysis (Figure S3).

### 2.3. Cell Culture

DC2.4 murine DC cells were a gift from Kenneth L. Rock [41]. Cells were grown at 37 °C in a humidified atmosphere containing 5% CO_2_. DC2.4 cells were grown in RPMI 1640 medium supplemented with 10% heat-inactivated fetal bovine serum. HEK293 human immortalized cells were grown in Eagle’s Minimum Essential Medium supplemented with 10% heat-inactivated fetal bovine serum. Cells were mycoplasma-free as evidenced by MycoAlert Mycoplasma Detection Kit (Lonza, Levallois Perret, France).

### 2.4. LNP Preparation

LNPs were prepared according to [42,43]. Briefly, D-Lin-MC3-DMA, DSPC, DOPE, cholesterol, and DMG-PEG-lipid were solubilized in ethanol at a molar ratio of 50:10:38.5:1.5, and a nitrogen-to-phosphate ratio of 6. mRNA was dissolved in citrate buffer (50mMcitrate buffer [pH 4.0]). The two components were mixed at a 3:1 volume ratio of mRNA to lipid solution with a 12 mL/min flow rate using an Ignite microfluidic system (Precision Nanosystems, Vancouver, Canada). mRNA-containing samples were diluted 40-fold with PBS (pH 7.4) and concentrated by centrifugation with Amicon^®^ Ultra-15 Centrifugal Filter Units (Merck, Lyon, France) at 4000 g for 30 min at 4 °C. The encapsulation efficiency was measured with the Quant-iT RiboGreen RNA Assay Kit (Life Technologies, Carlsbad, CA, USA) using a microplate reader.

### 2.5. LNP Characterization

The size and zeta potential of LNP were determined by DLS using Nano S zetasizer (Malvern) and, a SZ-100 nanoparticle analyzer (Horiba), respectively. LNPs were diluted in PBS (phosphate-buffered saline, pH 7.4) before DLS and zeta potential measurements. For aggregation studies, the size of LNPs was monitored after incubation at 37 °C with PBS containing 7% heat-inactivated fetal bovine serum, as in [38].

### 2.6. Gel Electrophoresis

LNPs and free mRNA were analyzed on a 1% agarose gel containing Ribogreen (ThermoFisher) at a 1/5000 dilution. 1 µg of mRNA was loaded per lane. Gels were imaged using a GelDocXR^+^ Imager (Biorad, Hercules, CA, USA).

### 2.7. Transfections and Flow Cytometry

Cells were transfected with either LNP or Lipofectamine Messenger Max (LFM, Thermo Fisher) as commercial standard using 1.7 µL of LFM per µg of mRNA in OptiMEM, at 70–80% confluency in 24-well plates using containing 0.5 μg of RNA encoding EGFP per well. Transfection was performed in complete media. Transfection efficiency was evaluated at 24 h after transfection. The cell-associated fluorescence intensity was measured with a flow cytometer (FORTESSA X20; Becton Dickinson, Franklin Lakes, NJ, USA) with λex = 488 nm; λem = 530 ± 30 nm. The fluorescence intensity was expressed as the mean fluorescence intensity of 10,000 events.

For cellular uptake experiments, LNP were prepared with Cyanine 3-labeled mRNA. mRNA was labeled with Cyanine 3 using Label IT^®^ Nucleic Acid Labeling Reagents (Mirus Bio LLC, Madison, WI, USA), following manufacturer’s instructions.

### 2.8. In Cellulo Cytotoxicity

Cytotoxicity was evaluated performing an MTT (3-(4,5-dimethylthiazol-2-yl)-2,5-diphenyltetrazolium bromide) assay as in [37]. Briefly, a solution of MTT (5 mg/mL in PBS) was added to the cells in a culture medium, and the cells were incubated for 4 h at 37 °C. Then, the formazan crystals were solubilized with acidic isopropanol and the absorbance was measured at 570 nm with a Victor I spectrophotometer. Cell viability was expressed as a percentage of the absorbance of untransfected cell cultures in the same conditions.

### 2.9. Analysis of Intracellular Distribution of LNP by Confocal Microscopy

We used confocal microscopy to evaluate the subcellular distribution of mRNA-LNPs using a LSM 980 Airyscan 2 confocal microscope (Carl Zeiss, Oberkochen, Germany), based on [44]. DC 2.4 cells were seeded on glass coverslips in 24-well plates at 120,000 cells per well. Early endosomes were stained by transduction with CellLight^®^ Early Endosomes-GFP baculoviruses expressing the early endosomes protein Rab5a fused to green fluorescent protein (Thermo Fisher Scientific Inc., Yokohama, Japan) following manufacturer’s protocol. To label other organelles, cells were transfected with pDNA encoding either EGFP-Rab7 (late endosomes) or EGFP-Rab11 (recycling endosomes). EGFP-Rab pDNA was transfected using Lipofectamine 3000 (ThermoFisher) with 0.5 µg mRNA per well 24 h before incubation with mRNA-LNP complexes. Cells were incubated with complexes prepared with 0.5 µg Cy3-labeled mRNA for 4 h at 37 °C. Then, cells were washed with PBS before fixation in 4% paraformaldehyde and mounted with Fluoromount-G™ Mounting Medium containing DAPI (Thermo Fischer, Waltham, MA USA). Weighted colocalization coefficients between Cy3-labeled mRNA and GFP- labeled organelles were determined using Zen Blue software version 3.2 (Carl Zeiss, Oberkochen, Germany). This calculation considers the intensity values of the summed pixels. The software uses the following equation for Pearson’s colocalization coefficient (PCC) calculation: $\mathrm{PCC}=\frac{\sum \left(Ch1i-Ch1avg\right)\left(Ch2i-Ch2avg\right)}{\surd (\sum {\left(Ch1i-Ch1avg\right)}^{2}{\left(Ch2i-Ch2avg\right)}^{2})}$. The calculation analyses take into account the signal intensities in colocalized and non-colocalized regions.

### 2.10. In Vivo mRNA Expression

All procedures were approved by the French Ministry of Research (#30279). 7–8 weeks Balb/c mice received 5 µg Cap1-capped and 5-methoxyuridine modified firefly luciferase mRNA (Oz Biosciences, Marseille, France) formulated as LNP. LNP were injected into the tibialis muscle. Animals were imaged at 6 h, 24 h, 48 h, and 72 h post-injection using an IVIS Lumina system Imaging system (PerkinElmer, Villebon-sur-Yvette, France) 5 min after intraperitoneal injection of 200µl of the D-Luciferin substrate (Promega, Madison, WI, USA). Luciferase signal was quantified using Living Image software (Perkin Elmer).

### 2.11. Statistical Analysis

The data were tested for statistical significance using ANOVA. All numerical data are expressed as mean ± SD, *n* = 3. Experiments were performed twice in triplicates. Any *p*-value less than 0.05 was considered statistically significant.

## 3. Results and Discussion

### 3.1. Formation of LNP

**Figure 1:**
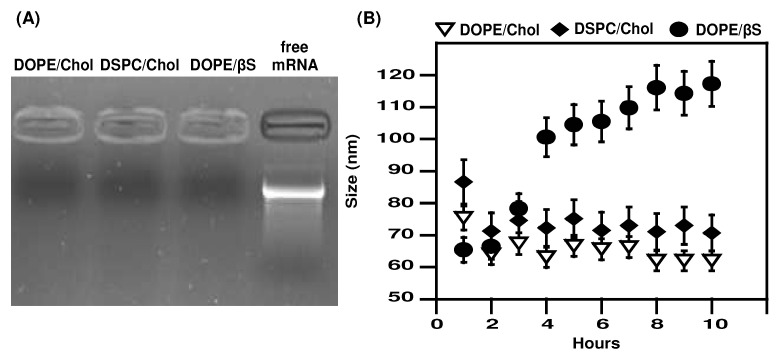
mRNA complexation and stability of mRNA LNPs in serum-containing media. (**A**), mRNA complexation: Free or LNP-complexed mRNA were run on an agarose gel electrophoresis; (**B**), Size stability: LNPs were incubated in media containing bovine serum at 37 °C and size was monitored by DLS. DLS measurements are the mean of two experiments.

Based on previous reports, we prepared LNPs with the following molar ratios: 50% of D-Lin-MC3 ionizable lipid, 10% helper lipid (DSPC or DOPE), 38.5% of sterol (cholesterol or β-sitosterol) and 1.5% PEGylated lipid at an N/P ratio of 6 [9,10,33]. Structures of lipids are presented in Figure S1. This resulted in testing three formulations: MC3/DSPC/Cholesterol/PEG (referred as DSPC/Chol), MC3/DOPE/Cholesterol/PEG (referred as DOPE/Chol), and MC3/DOPE/β-sitosterol/PEG (referred as DOPE/βS). βS possesses an additional ethyl group at the C24 position. All formulations resulted in LNPs of 76–95 nm with a neutral zeta potential (Table 1). Complexation of mRNA was verified by gel electrophoresis, with no free mRNA detectable (Figure 1A). Interestingly, DSPC containing LNPs exhibited a lower polydispersity index (0.066 for MC3/DSPC/Cholesterol/PEG vs. 0.16 for MC3/DOPE/Cholesterol/PEG and 0.13 for MC3/DOPE/β-sitosterol/PEG). These results indicate similarly low polydispersity for the three types of LNPs. We also monitored the size of LNPs after incubation with serum and at 37 °C by dynamic light scattering (Figure 1B). Proteins in serum could interact with the surface of LNPs and induce their aggregation. The three types of LNPs remained stable during incubation confirming the PEG-mediated stabilization of the nanoparticles in serum as previously reported [38,45,46]. The size of DOPE/βS LNPs moderately increased from 90 nm to 120 nm over 8 h. This may suggest an evolution (a rearrangement/fusion of LNP) toward larger particles induced by the presence of βS at the LNP surface, as it has been observed for liposomes [47]. Nevertheless, a moderate 20–30 nm size increase of mRNA-LNPs in the presence of serum was also reported by Miao et al. suggesting this will not affect the capacity of DOPE/βS LNPs to transfect cells [46].

### 3.2. Cellular Transfection Efficiency

**Figure 2:**
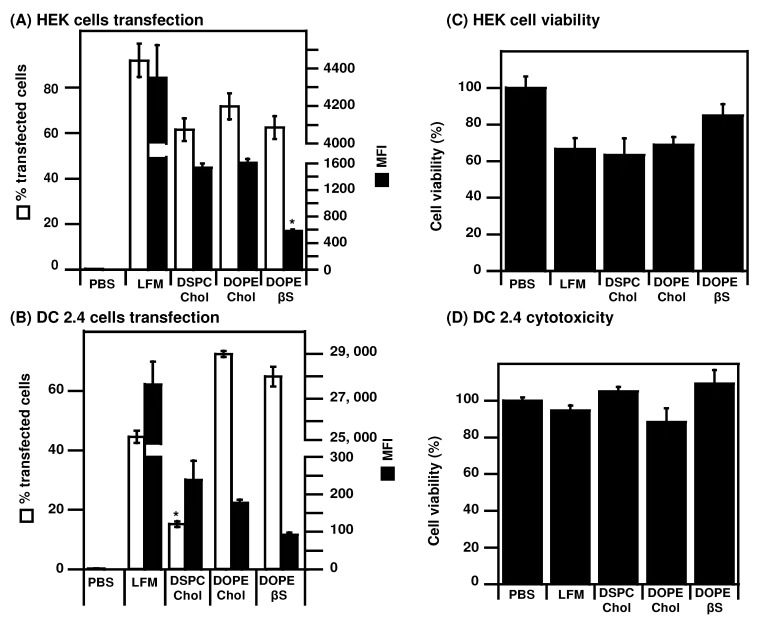
Evaluation of mRNA LNPs transfection efficiency: LNPs prepared with EGFP mRNA were used to transfect HEK cells (**A**,**C**) or DC 2.4 cells (**B**,**D**). Transfection efficiency and cytotoxicity were measured 24 h after transfection. Data are presented as mean ± SD of two experiments performed in triplicates, * *p* < 0.05 compared to other LNPs.

Next, we compared the transfection efficiency of the three types of LNPs (Figure 2).

As most in cellulo studies on mRNA LNPs use human cancer cell lines or immortalized cells [20,35,48], we also transfected human HEK cells (Figure 2A,C). In these immortalized cells, the three types of LNPs were capable of transfecting more than 60% of cells, albeit lower than the commercial LFM standard (90% transfected cells). Contrary to DCs, lower MFI were detected in HEK cells transfected with DOPE/βS LNPs (583 MFI) compared to DSPC/Chol LNPs (1539 MFI) and DOPE/Chol LNPs (1607). In HEK cells, their transfection with LFM, DOPE/Chol LNPs, and DSPC/Chol LNPs induced cytotoxicity leading to only 65–70% viable cells after transfection (Figure 2C). Interestingly, DOPE/βS LNPs were better tolerated with 80% viable cells after transfection.

As our focus is mRNA vaccination, we also evaluated the transfection efficiency of those formulations on the murine DC2.4 DC cell line, [49,50,51] (Figure 2B,D). The percentage of transfected DC is lower (40–70%) compared to that of HEK cells (60–80%). Lower transfection efficiency in murine dendritic cells was expected as they are notoriously harder to transfect than human cancer or immortalized cells [52,53] but it could also be attributed to the species-dependent cellular response to mRNA LNPs recently reported [54]. Whereas DOPE/Chol and DOPE/βS LNPs transfected 65–70% DCs—that is more than the commercial standard Lipofectamine Messenger Max^®^ (LFM, 45%)—DSPC/Chol LNPs only transfected 15% of DCs. When looking at the mean fluorescence intensity (MFI), correlated with intracellular eGFP copies related to mRNA expression, a striking contrast is observed between LFM (28,000 MFI) and LNPs (90–240 MFI). Better transfection efficiency of DOPE-LNPs over DSPC-LNPs is in agreement with previous optimization experiments made for mRNA LNPs [22,30,33]. Such a gap in MFI, corresponding to a 100-fold difference, suggests different intracellular processing of the two types of complexes in DCs. Note that more than 80% of cells were viable in all groups after transfection indicating a lack of cytotoxicity (Figure 2D).

### 3.3. Intracellular Trafficking of mRNA in DCs

**Figure 3:**
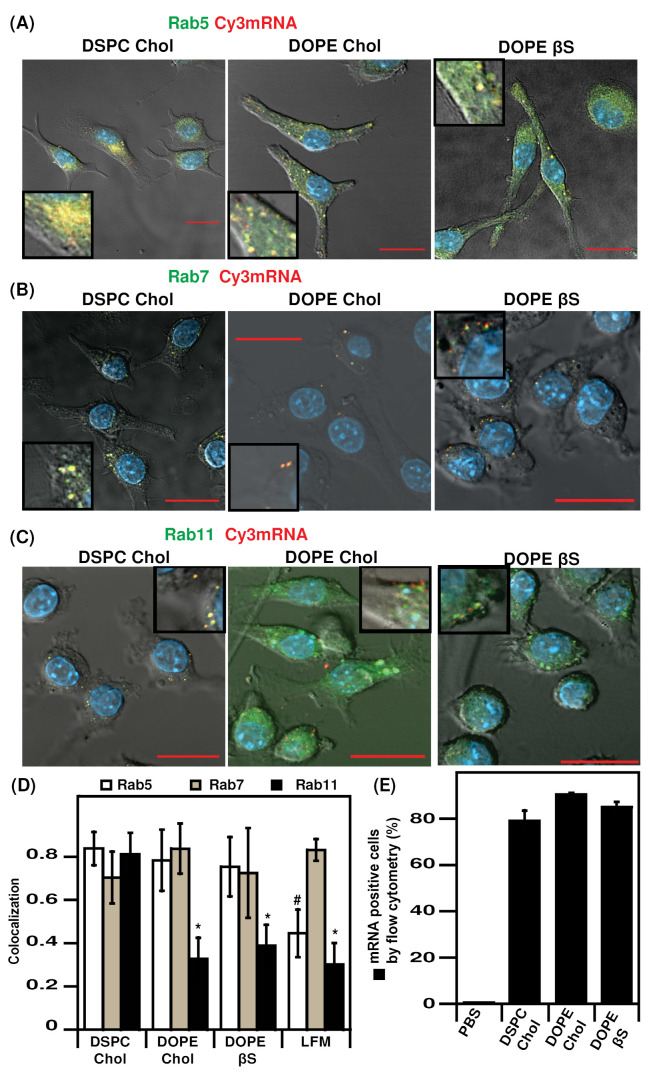
Intracellular trafficking of Cy3 mRNA-LNPs in DC 2.4 cells by confocal microscopy and flow cytometry: (**A**), Rab5-GFP colocalization with Cy3 mRNA; (**B**), Rab5-GFP colocalization with Cy3 mRNA; (**C**), Rab5-GFP colocalization with Cy3 mRNA; (**D**), Pearson’s colocalization coefficient quantification; (**E**), quantification of Cy3 mRNA-LNPs uptake in DC 2. cells by flow cytometry. Nuclei were stained with DAPI. Bar represents 20 µm. Data are presented as mean ± SD, * *p* < 0.05 compared to DSPC/Chol LNPs., # *p* < 0.05 compared to all LNPs. Zoomed areas are shown in inserts.

We deciphered the intracellular fate of the three types of LNPs in DC. To do so, we combined quantitative cellular uptake by flow cytometry and confocal microscopy. The three types of LNPs exhibited a similar cellular internalization with more than 80% cyanin3 mRNA-positive cells after 4 h incubation (Figure 3E), in agreement with previous studies [22,42].

We further investigated the intracellular trafficking of Cy3-labeled mRNA using confocal microscopy and cells expressing chimera fluorescent proteins to label critical organelles: EGFP-Rab5, EGFP-Rab7, and EGFP-Rab11 to label early endosomes, late endosomes, and recycling endosomes, respectively (Figure 3A–C and Figure S2) [16,55,56,57]. Endosomal trafficking was studied 4 h after transfection as in the original report comparing cholesterol and β-sitosterol LNPs [21]. A summary of Pearson’s colocalization coefficient (PCC) data is presented in Figure 3D, representative confocal pictures for each condition are presented in Figure 3A–C. The three types of LNPs exhibited high retention in early endosomes, with PCCs of 0.7–0.8. By contrast, LFM had a markedly decreased retention in early endosomes (PCC of 0.44) suggesting a faster intracellular routing (Figure S2).

Interesting to note that the percentage of transfected cells by DOPE/Chol and DOPE/βS formulations is higher than that of LFM. By contrast, the translation efficiency reflected by the MFI values (Figure 2) of cells transfected with LFM is at least 100-fold more than those obtained with other LNPs. Therefore, it is tempting to suggest that the low retention of LFM formulations in early endosomes could be due to a better endosomal escape of LFM-delivered mRNA leading to the higher mRNA expression with LFM in DCs.

The three types of LNPs and LFM resulted in a similar mRNA distribution in late endosomes. Such a lack of difference in Rab7-compartments trafficking of nanoparticles with different transfection efficiency agrees with a previous study on LNP intracellular trafficking indicating a lack of predictive value of late endosome distribution [58]. Furthermore, the indistinguishable endosomal distribution profiles of cholesterol and β-sitosterol LNPs are in agreement with a previous report [21].

We also imaged the distribution of mRNA in Rab11 recycling endosomes which play a key role in routing to exocytosis or lysosomes [59,60,61]. LFM, DOPE/Chol, and DOPE/βS which are the most efficient formulations in DC2.4 cells in terms of percentage of transfected cells had a comparably low distribution in Rab11 compartments with PCCs between 0.3 and 0.4. On the contrary, DSPC/Chol LNPs which transfected less than 20% DC2.4 were highly localized in recycling endosomes (PCC or 0.81). This observation suggests that those particles could be exocytosed explaining the low transfection efficiency. Such an inverse correlation between Rab11 accumulation and transfection efficiency in dendritic cells is consistent with results on DNA lipoplexes in human cancer cells [62].

Last, the difference in transfection efficiency cannot be due to the difference in endocytosis. Indeed, the intracellular quantification of Cy3-mRNA delivered with the different formulations revealed that all formulations are similarly up taken by the DC2.4 cells.

### 3.4. In Vivo Expression of mRNA

**Figure 4:**
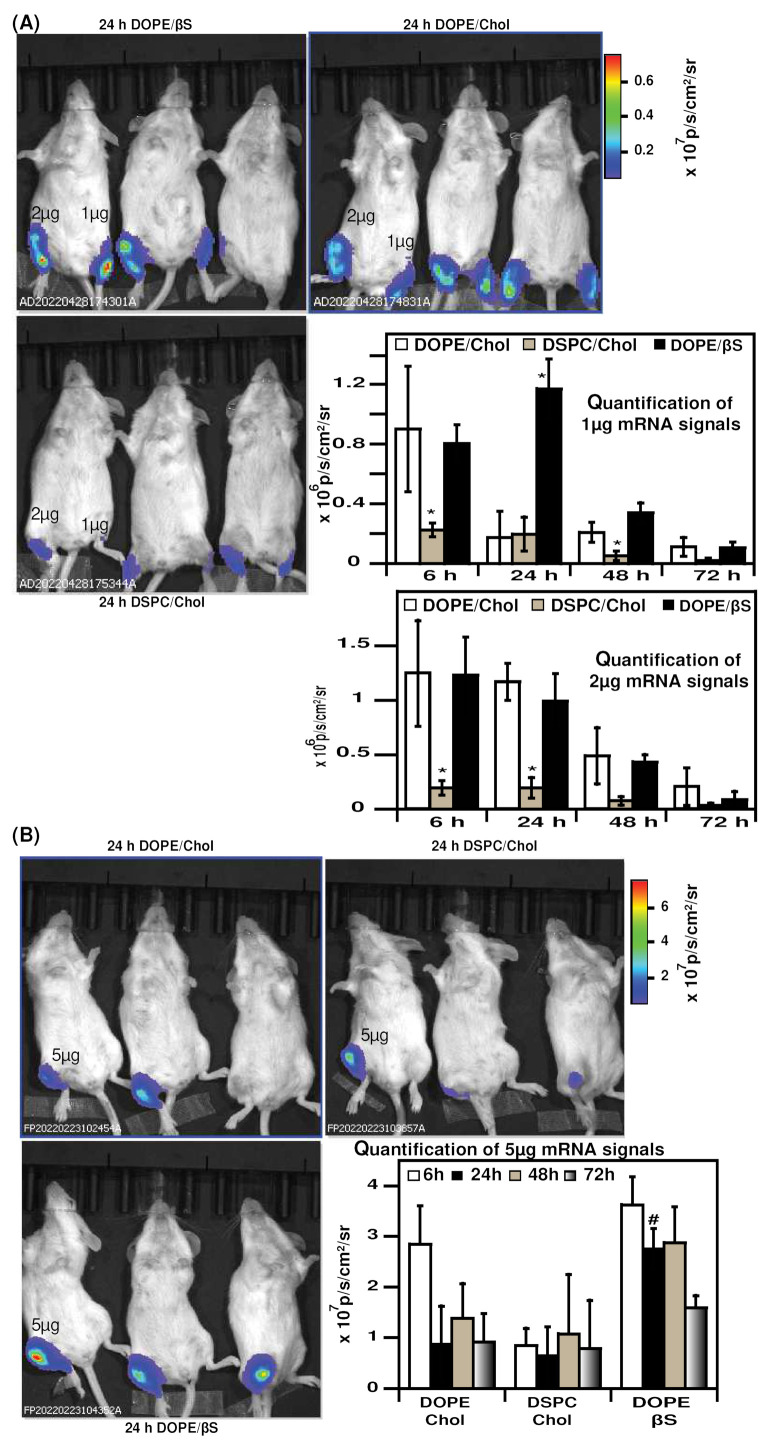
Evaluation of mRNA LNPs in vivo expression: (**A**), representative images of luciferase expression 24 h after intramuscular injection of 1 µg or 2 µg mRNA-LNPs, signal was during 3 days (*n* = 3); (**B**), representative images of luciferase expression 24 h after intramuscular injection of 5 µg mRNA-LNPs, signal was during 3 days (*n* = 3). Data are presented as mean ± SD, * *p* < 0.05 compared to DOPE/Chol LNPs and DOPE/βS LNPs, # *p* < 0.05 compared to DOPE/Chol and DSPC/Chol LNPs.

We next tested the expression of mRNA LNPs in mice. We chose the intramuscular route as it allows a maximal localized mRNA expression and it was the route used for the approved mRNA vaccines [9,10,11,53]. To the best of our knowledge, no study compared DSPC/Chol, DOPE/Chol, and DOPE/βS LNPs in vivo. We compared three doses of mRNA: 1 µg, 2 µg and 5 µg mRNA as low, intermediate, and high dose, respectively (Figure 4). Noteworthy, when the lowest dose of mRNA-LNPs was injected (Figure 4, mouse’s left paws), DOPE/βS LNPs achieved superior luciferase expression over DOPE Chol and DSPC/Chol LNPs at 24 h (6.7 and 6-fold, respectively).

When formulated with 2 µg mRNA (Figure 4, mouse’s right paws), DOPE/βS and DOPE/Chol NPs resulted in similar expression from 6 h to 72 h. Whilst DSPC/Chol LNPs were inferior with a five-fold higher luciferase signal compared to DOPE/βS and DOPE/Chol NPs. Following the same tendency, injection of 5 µg mRNA (Figure 4B) produced a similar signal with DOPE/βS and DOPE/Chol LNPs at 6 h, 48 h, and 72 h. At 24 h post-injection, DOPE/βS LNPs resulted in the brightest luminescence: three-fold over DOPE/Chol LNPs and DSPC/Chol LNPs. DSPC/Chol group showed the lowest luciferase signal at 6 h and 24 h confirming the results obtained with 1 µg and 2 µg mRNA doses.

We must point out that studies comparing DOPE and DSPC co-lipids in mRNA LNPs have been performed with intravenous or subcutaneous injection routes rather than intramuscular routes [22,30,33]. Our experiments confirmed the previous studies with the benefit of DOPE and βS combination for in vivo mRNA delivery, particularly evident at 1 µg and 5 µg doses of mRNA. In accordance with our study, Alvarez-Benedicto et al. reported better expression of luciferase in the spleen and liver of luciferase after intravenous injection in mice of mRNA LNPs containing DOPE over DSPC [22].

Our results of better efficacy of DOPE-containing LNPs over DSPC-containing LNPs might be explained by differences in corona formation and cell interaction with DSPC and DOPE-LNPs for each route. Yet, complementary studies will be required to decipher the impact of cholesterol, β-sitosterol and DOPE on the structure of LNPs. Such multiscale analyses including cryo-TEM and SAXS will connect LNP structure and lipid location with LNP activity in cellular models and in vivo. Oberli et al. evidenced as well an induction of inflammation at the injection site after subcutaneous injection of DSPC-LNPs in 20% of mice and 0% of mice with DOPE-LNPs suggesting an impact of the helper lipid on immune cell activation/recruitment in vivo [33]. The findings reported are in accordance with those of Hattori et al. on sterol derivatives for siRNA delivery in murine cancer cells and mice lungs [63]. They compared the silencing activities of lipoplexes made of DOTAP (1,2-dioleoyl-3-trimethylammonium-propane) and four different sterol derivatives: cholesterol, stigmasterol, ergosterol, and βS. DOTAP-based lipoplexes moderately inhibited gene expression irrespective of the sterol derivative in murine cancer cells. They noted similar gene knockdown in the liver 48 h after intravenous injection of 20 µg siRNA –reflecting a high dose of RNA- delivered by cholesterol or βS liposomes, corroborating the comparable luciferase expression we observed after intramuscular injection of 5 µg cholesterol or βS mRNA LNPs at 48 h.

## 4. Conclusions

We present here the first study comparing both DOPE or DPSC and cholesterol or β-sitosterol for mRNA delivery in cellulo and in vivo.

Previous experiments comparing β-sitosterol and cholesterol were performed in HEK293 human cells only and no data in immune cells nor in vivo have been reported yet. They reported a 20-fold higher expression of mRNA after transfection with β-sitosterol LNPs over cholesterol LNPs in HEK293 cells. However, using EGFP mRNA as a reporter, we observed similar percentages of transfected cells with LNPs prepared with cholesterol or its analog in HEK cells and murine dendritic cells. The levels of cell-associated fluorescence were even lower for β-sitosterol LNPs compared to cholesterol LNPs suggesting a difference in translation activity in both cell lines. The benefit of DOPE and βS was more evident in vivo and particularly at the lower mRNA dose of 1 µg mRNA with higher reporter mRNA expression at 24 h.

Altogether this study provides valuable information for helper lipid choice for dendritic cell transfection and intramuscular administration of mRNA-LNPs vaccines.

## Figures and Tables

**Table 1:** Size and zeta potential of LNPs.

Composition	Size (nm)	Polydispersity Index	Zeta Potential (mV)
MC3/DOPE/Chol/PEG	84/13	0.16/0.02	−1.6/2.3
MC3/DSPC/Chol/PEG	76/6	0.066/0.046	4/1.5
MC3/DOPE/βS/PEG	95/5	0.13/0.1	−0.1/3
Lipofectamine Messenger max	240/46	0.41/0.22	−1.6/3

Abbreviations: Chol, cholesterol; βS, β-sitosterol. *n* = 3, data are presented with standard deviation.

## Data Availability

The data presented in this study are available on request from the corresponding author.

## Supplementary Materials

The following supporting information can be downloaded at: https://www.mdpi.com/article/10.3390/nano12142446/s1, Figure S1: Structures of the lipids used in this study; Figure S2. Intracellular trafficking of Cy3 mRNA-LFM complexes in DC 2.4 cells by confocal microscopy with either Rab5-GFP, Rab7-GFP or Rab11-GFP, Figure S3: Agilent analysis of the GFP mRNA produced by in vitro transcription.
