# Supplementary material for: In Cellulo and In Vivo Comparison of Cholesterol, Beta-Sitosterol and Dioleylphosphatidylethanolamine for Lipid Nanoparticle Formulation of mRNA
Source: Nanomaterials (Basel). 2022 Jul 17;12(14):2446. doi: 10.3390/nano12142446 (PMC9317807; doi:10.3390/nano12142446)
Supplement: Supplementary file 1 [file nanomaterials-12-02446-s001.zip › nanomaterials-1808062-supplementary.pdf]

Supplementary Information for

In Cellulo and In Vivo Comparison of Cholesterol, Beta-Sitosterol and Dioleylphosphatidylethanolamine for Lipid Nanoparticle Formulation of mRNA

Ayoub Medjmedj [1,†], Albert Ngalle-Loth [1,†], Rudy Clemençon [1], Josef Hamacek [1,2], Chantal Pichon [1,2] and Federico Perche [1,*]

[1] Centre de Biophysique Moléculaire UPR4301 CNRS, Rue Charles Sadron, 45071 Orléans, France; ayoub.medjmedj@cnrs-orleans.fr (A.M.); albert.ngalle-loth@cnrs-orleans.fr (A.N.-L.); rudy.clemencon@cnrs.fr (R.C.); josef.hamacek@cnrs.fr (J.H.); pichon@cnrs.fr (C.P.)
[2] Centre de Biophysique Moléculaire, University of Orléans, 45100 Orléans, France
* Correspondence: federico.perche@cnrs-orleans.fr; Tel.: +33-2-38-25-55-44
† These authors contributed equally to this work.

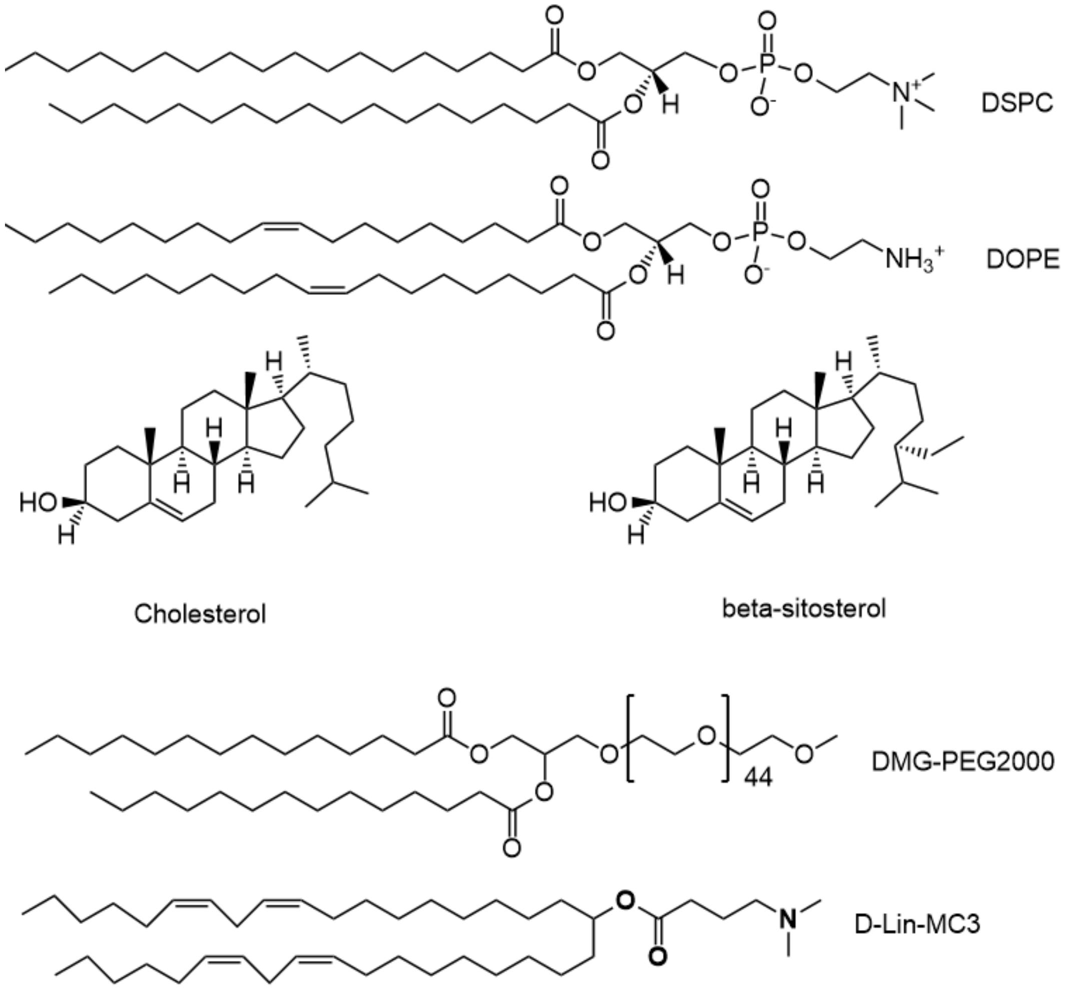

Figure S1. Structures of the lipids used in this study.

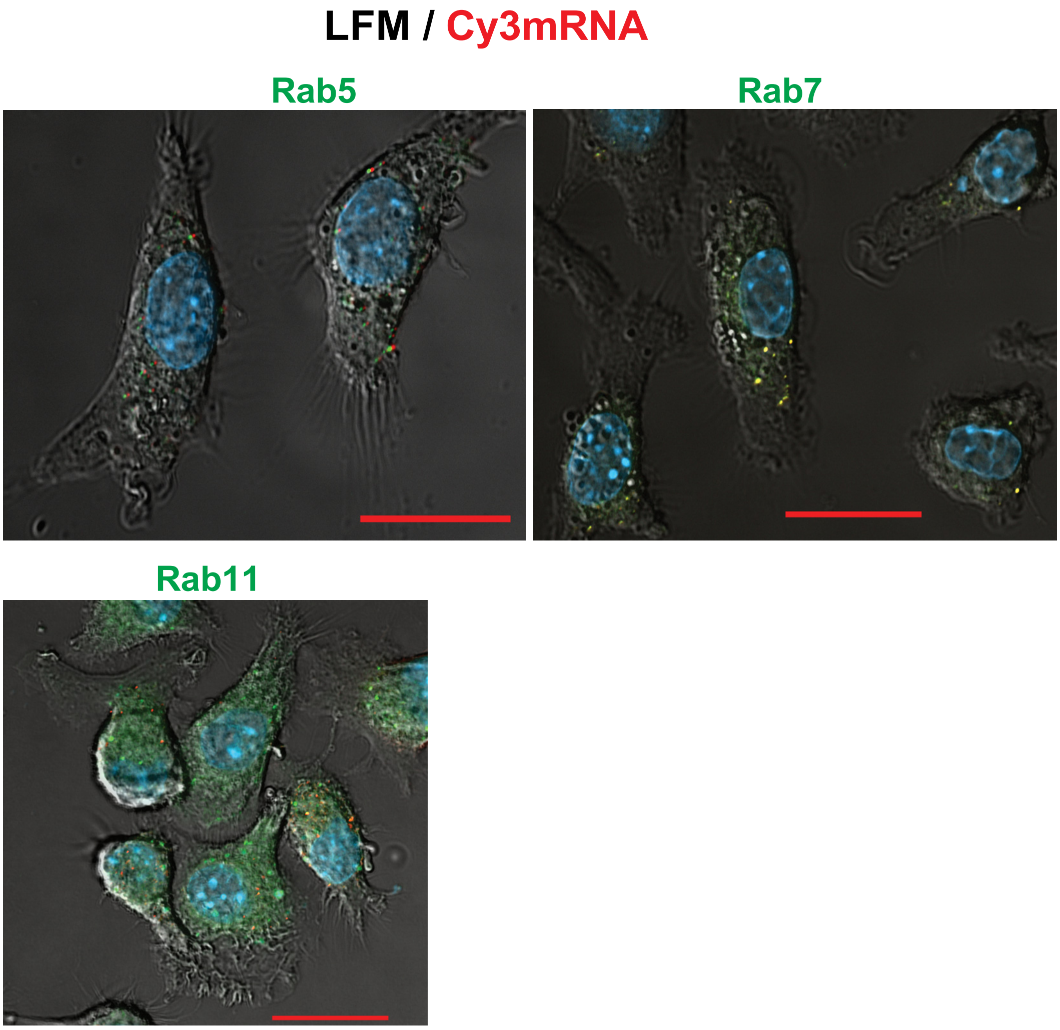

Figure S2. Intracellular trafficking of Cy3 mRNA-LFM complexes in DC 2.4 cells by confocal microscopy with either Rab5-GFP, Rab7-GFP or Rab11-GFP. Nuclei were stained with DAPI. Bar represents 20 µm.

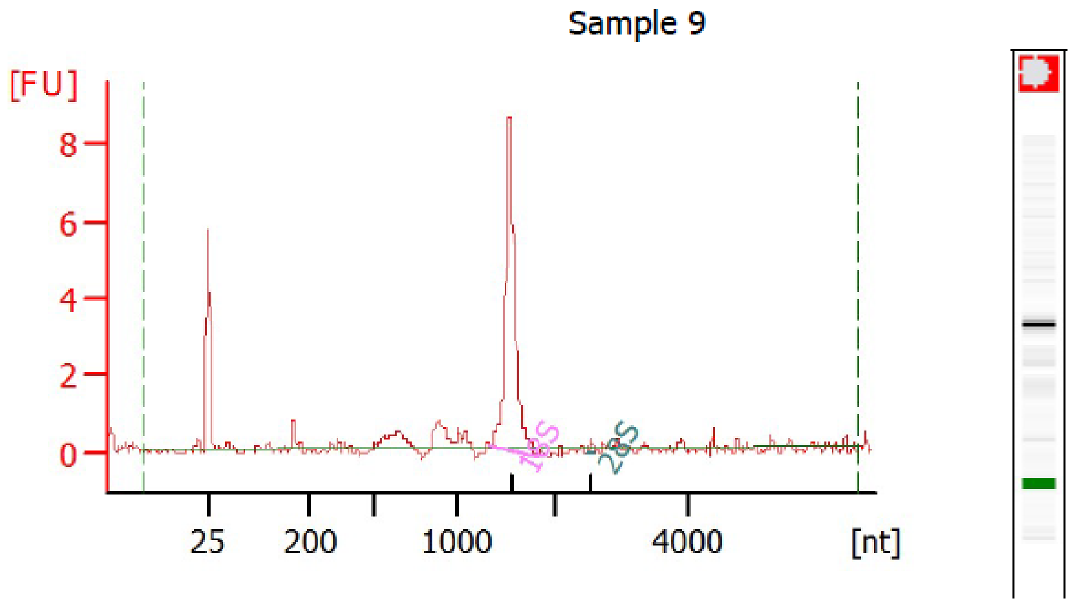

Figure S3. Agilent analysis of the GFP mRNA produced by *in vitro* transcription.
